# Is lymphovascular invasion a powerful predictor for biochemical recurrence in pT3 N0 prostate cancer? Results from the K-CaP database

**DOI:** 10.1038/srep25419

**Published:** 2016-05-05

**Authors:** Yong Hyun Park, Yejin Kim, Hwanjo Yu, In Young Choi, Seok-Soo Byun, Cheol Kwak, Byung Ha Chung, Hyun Moo Lee, Choung Soo Kim, Ji Youl Lee

**Affiliations:** 1 Department of Urology, Seoul St. Mary’s Hospital, The Catholic University of Korea College of Medicine, Seoul, Korea; 2 Department of Creative IT Engineering, Pohang University of Science and Technology, Pohang, Korea; 3 Department of Medical Informatics, The Catholic University of Korea College of Medicine, Seoul, Korea; 4 Department of Urology, Seoul National University Bundang Hospital, Seongnam, Korea; 5 Department of Urology, Seoul National University Hospital, Seoul, Korea; 6 Department of Urology, Yonsei University Health System, Seoul, Korea; 7 Department of Urology, Samsung Medical Center, Sungkyunkwan University School of Medicine, Seoul, Korea; 8 Department of Urology, Asan Medical Center, University of Ulsan College of Medicine, Seoul, Korea

## Abstract

To assess the impact of lymphovascular invasion (LVI) on the risk of biochemical recurrence (BCR) in pT3 N0 prostate cancer, clinical data were extracted from 1,622 patients with pT3 N0 prostate cancer from the K-CaP database. Patients with neoadjuvant androgen deprivation therapy (n = 325) or insufficient pathologic or follow-up data (n = 87) were excluded. The primary endpoint was the oncologic importance of LVI, and the secondary endpoint was the hierarchical relationships for estimating BCR between the evaluated variables. LVI was noted in 260 patients (21.5%) and was significantly associated with other adverse clinicopathologic features. In the multivariate Cox regression analysis, LVI was significantly associated with an increased risk of BCR after adjusting for known prognostic factors. In the Bayesian belief network analysis, LVI and pathologic Gleason score were found to be first-degree associates of BCR, whereas prostate-specific antigen (PSA) level, seminal vesicle invasion, perineural invasion, and high-grade prostatic intraepithelial neoplasia were considered second-degree associates. In the random survival forest, pathologic Gleason score, LVI, and PSA level were three most important variables in determining BCR of patients with pT3 N0 prostate cancer. In conclusion, LVI is one of the most powerful adverse prognostic factors for BCR in patients with pT3 N0 prostate cancer.

Prostate cancer is the most common newly diagnosed cancer in males, and the second most common cause of cancer-related death in the United States^1^. Approximately 25–50% of patients undergoing radical prostatectomy harbor extracapsular disease^2^, which is associated with an increased risk of biochemical recurrence (BCR)^3^,^4^. Recently published studies have demonstrated that adjuvant treatment for selected patients can reduce the risk of BCR^5^,^6^; however, there are currently several important issues concerning this, particularly regarding which patients will benefit from and should be offered adjuvant treatment.

The prognosis of patients with prostate cancer is currently assessed by the TNM staging system after radical prostatectomy. However, the 5-year BCR-free survival for pT3 patients has been reported to range widely (10–66%) according to the presence or absence of a variety of histopathologic, biological, and patient factors^7^. Lymphovascular invasion (LVI) has been demonstrated to be an independent predictor of poor prognosis in several solid tumors^8^,^9^,^10^. Because cancer cells must adhere, penetrate, and migrate into the blood or lymphatic vessels before entering circulation^11^, LVI is believed to be associated with a predisposition for disease recurrence or distant metastasis. In a previous study on clinically localized prostate cancer, LVI was demonstrated to be associated with aggressive disease and BCR^12^,^13^, although it was concluded that it may not be useful for improving the already established predictive models^13^. Additionally, several small series have reported LVI as an independent predictor of disease recurrence in patients with pT3 N0 prostate cancer^14^,^15^,^16^; however, there is still some controversy regarding its prognostic significance in prostate cancer^13^.

Therefore, the purpose of the current study was to demonstrate the hierarchical relationships between various variables, including LVI, for estimating BCR; and to assess the impact of LVI on the risk of BCR in patients with pT3 N0 prostate cancer treated with radical prostatectomy.

## Results

### Baseline Demographics

The baseline characteristics of the 1,210 study patients are presented in Table 1. The mean age was 66.2 years, and the mean serum prostate-specific antigen (PSA) level was 15.7 ng/mL. LVI was noted in 260 patients (21.5%) and was significantly associated with several other adverse clinicopathologic features, such as high preoperative PSA level, large tumor volume, positive surgical margin, seminal vesicle invasion (SVI), perineural invasion, and high pathologic Gleason score. Postoperative adjuvant treatment was performed in 219 patients (18.1%) as follows: radiation therapy and androgen deprivation therapy (n = 100, 8.3%), radiation therapy only (n = 79, 6.5%), and androgen deprivation therapy only (n = 40, 3.3%).

### Prognostic Importance of LVI

**Figure 1:**
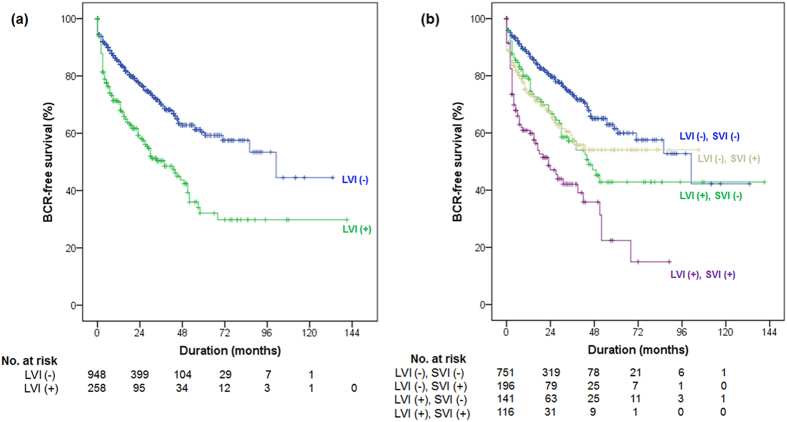
(**a**) BCR-free survival according to LVI in the K-CaP cohort (p < 0.001). (**b**) BCR-free survival according to LVI and SVI in the K-CaP cohort (p < 0.001).

At a median follow-up of 32.0 months from radical prostatectomy, BCR was observed in 352 (29.1%) patients, including 232 (24.4%) patients without LVI and 120 (46.3%) patients with LVI (p < 0.001). The 5-year BCR-free survival rates were 60.3% and 32.1% in patients without and with LVI, respectively (log rank test, p < 0.001, Fig. 1). When the patients were stratified into 4 groups based on the LVI and SVI status, the 5-year BCR-free survival rates were 61.5% in patients without LVI and SVI (n = 752), 54.1% in those with SVI only (n = 198), 42.8% in those with LVI only (n = 143), and 22.4% in those with both LVI and SVI (n = 117) (log rank test, pooled over strata, p < 0.001, Fig. 1). However, no significant difference in BCR-free survival was observed between patients with LVI only and SVI only (log rank test, pairwise over strata, p = 0.745).

In the univariate Cox proportional hazard regression analysis, LVI was significantly associated with an increased risk of BCR, and this association remained significant after adjusting for various known prognostic factors in the multivariate Cox proportional hazard regression analysis (Table 2).

**Figure 2:**
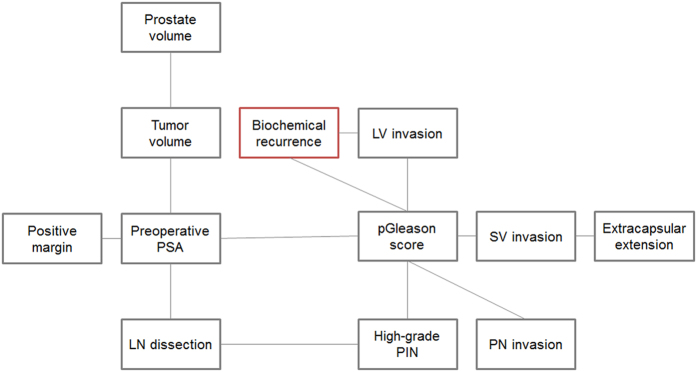
Bayesian belief network analysis for biochemical recurrence in the K-CaP cohort. PSA, prostate-specific antigen; LN, lymph node; LV, lymphovascular; pGleason, pathologic Gleason score; PIN, prostatic intraepithelial neoplasia; SV, seminal vesicle; PN, perineural.

Graphic structures of the Bayesian belief network analysis are displayed in Fig. 2. Bayesian belief network analysis revealed hierarchical associations between the various clinicopathologic features. As shown in Fig. 2, there were two first-degree associates of BCR, namely LVI and pathologic Gleason score. Second-degree associates of BCR included preoperative PSA level, SVI, perineural invasion, and high-grade prostatic intraepithelial neoplasia.

**Figure 3:**
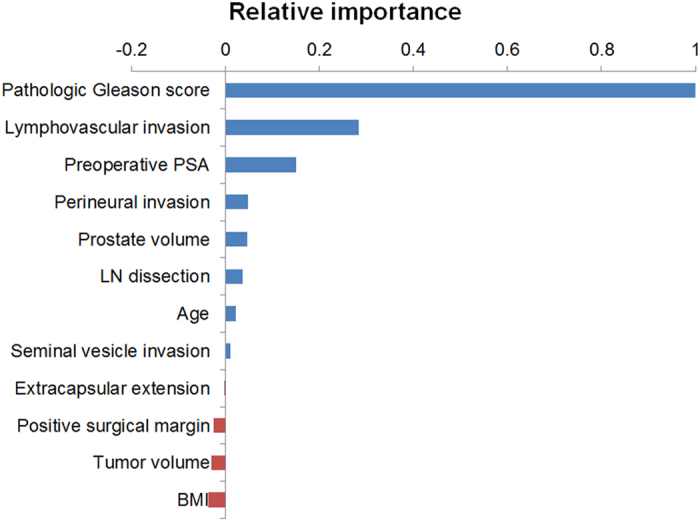
Relative importance of each clinicopathologic variable for BCR based on random survival forest analysis in the K-CaP cohort. PSA, prostate-specific antigen; LN, lymph node; BMI, body mass index.

Figure 3 shows the relative importance of each clinicopathologic feature to BCR based on the random survival forest analysis. The prediction model of random survival forest had the accuracy (C-index) of 0.76 ± 0.0005. Pathologic Gleason score (1.0), LVI (0.2836), and preoperative PSA level (0.1502) were found to be the three most important clinicopathologic factors in determining the risk of BCR in the patients with pT3 N0 prostate cancer. Conversely, the relative importance of SVI and positive surgical margin was only 0.0102 and −0.0248, respectively.

## Discussion

In the present study, LVI was identified in 21.5% of patients with pT3 N0 prostate cancer after radical prostatectomy. Additionally, LVI was also found to be associated with several adverse clinicopathologic features and poor BCR-free survival. However, there appear to be substantial inconsistencies regarding the association between LVI and BCR after radical prostatectomy in the literature. Ng and colleagues performed a systematic review in this subject and reported that only 58% of the included studies found that LVI was as an independent predictor of BCR with odds ratios or relative risks of 1.39–4.39^17^. However, many of these previous studies included patients with pathologically organ-confined prostate cancer (pT2 N0), which, due to the relatively low incidence of LVI and BCR in this population, may have resulted in bias. On the other hand, Yamamoto *et al.*^16^ reported that LVI was a significant predictor of BCR in 94 patients with pT3a N0 prostate cancer. Similarly, You *et al.*^18^ analyzed the prognostic factors of 397 patients with pathologic stage T3–T4 N0 disease and reported that LVI was observed in 18.6% of the patients and was an independent predicting factor of BCR in T3a disease. These results are in keeping with our results. However, in contrast to the present study, none of these prior studies have added LVI to the risk stratification for BCR.

There are a number of challenges in identifying LVI in prostatectomy specimens. Overdiagnosis due to mimickers is important challenges relating to the diagnosis of LVI in prostate cancer. Kryvenko *et al.*^19^ reported that retraction artifact, pseudoembolus, and ingrowth of myofibroblasts into large extraprostatic vessels are common mimickers of LVI. They also reported that there were some cases with LVI that were missed on routine hematoxylin and eosin sections. Although there might be several challenges and interobserver variability, LVI in prostatectomy specimens has been reported widely by experienced uropathologist. The International Collaboration on Cancer Reporting developed structured protocols for radical prostatectomy specimens, and considered that LVI should be listed as a recommended element^20^. They mentioned that diagnosis of LVI on hematoxylin and eosin sections is reliable, and immunohistochemical staining is rarely recommended.

Herein, we confirmed the prognostic significance of LVI in pT3 N0 prostate cancer using three different statistical methods. First, multivariate Cox proportional hazard regression analysis was performed to model the relationship between BCR-free survival and the different covariates. In the multivariate Cox proportional hazard regression analysis, several covariates, including LVI and SVI, were identified as independent predicting factors for BCR. However, the Cox proportional hazard regression analysis is not adept at assessing the relative importance of these factors or for discovery of potential interactions. Thus, to confirm the results of the Cox model and to assess the relationships between variables unexplained by this model, Bayesian belief network and random survival forest analyses were performed. These analyses were chosen for a variety of reasons. First, the random survival forest analysis is known to address the problems of restrictive assumption, such as the proportional hazards, of the Cox model^21^. Second, the random survival forest analysis has the advantages of intuitive comparisons of predictive importance between variables, and adjustment for potential multiple interactions in estimating importance^21^. Finally, the Bayesian belief network analysis provides a method for representing causal probabilistic relationships between variables by its graphical nature^22^. This allows the physicians or researchers to better understand the hierarchy and relative importance of each variable. In our study, all of the above statistical methods found that LVI was one of the most important risk factors for BCR in patients with pT3 N0 prostate cancer.

In our study, the impact of positive surgical margin on BCR failed to reach statistical significance in the multivariate Cox proportional hazard regression and random survival forest analyses. Many prior studies have reported that positive surgical margin is an independent predictive factor of BCR after radical prostatectomy, and it is considered as one of the most important factors to determine adjuvant treatment^23^. These discordances between the present and prior studies might have been caused by inclusion of patients with SVI in our study cohort. Several studies have reported that positive surgical margin is an independent factor in pT2 and T3a prostate cancer, but not in pT3b cancer^24^,^25^. Hence, we speculate that LVI, which is a pathologic variable reflecting potential for micrometastasis, is perhaps more important than positive surgical margin, which might be dependent on the local extent of the disease, in locally advanced cancers.

Our study has several important strengths and weaknesses. This is the non-randomized, retrospective study, which inherently associated with selection bias. Also, several pathologists from different institutes were involved during the study period without central pathology review. Nonetheless, it has the strengths of including a large number of pT3 N0 prostate cancer patients retrieved from the K-CaP database, an observational longitudinal multicenter database of Korean prostate cancer patients. Moreover, our study also suggested a possible prognostic role of LVI in pT3 N0 prostate cancer through novel statistical analysis. These novel statistical analytic methods could rectify several shortcomings of the traditional Cox proportional hazard regression analysis and provide evidence for the prognostic importance of LVI. The inclusion of LVI as a prognostic factor may improve the ability to inform pT3 N0 prostate cancer patients about their prognosis, and could be used to guide the decision for postoperative management in this population through the proper stratification of patients.

## Methods

### K-CaP Database Construction

Clinical information was extracted from the K-CaP database, an observational longitudinal database of Korean patients with biopsy-proven prostate cancer enrolled from 5 hospitals throughout Korea. Details regarding the K-CaP database have been previously reported^26^. The K-CaP database system provides 220 items for prostate cancer research; demographics, medical history, clinical information, pathologic results, follow-up data, and so on^27^.

### Eligible Patients and Outcomes

A total of 1,622 patients with pT3 N0 prostate cancer treated with radical prostatectomy between 2001 and 2012 were identified. To ensure a uniform population for evaluating the pathologic findings, patients were excluded if they received neoadjuvant androgen deprivation therapy (n = 325, 20.0%) or if they did not have sufficient pathologic or follow-up data (n = 87, 5.4%), resulting in a total of 1,210 patients being included in this study. LVI was defined as the presence of cancer cells within an arterial, venous, or lymphatic lumen on routine hematoxylin and eosin sections. The primary endpoint was to assess the oncologic importance of LVI on the risk of BCR in patients with pT3 N0 prostate cancer. The secondary endpoint was to determine the hierarchical relationships between variables for estimating BCR. Clinical outcome measures included age, body mass index, serum PSA level, biopsy Gleason score, clinical stage, pathologic Gleason score, pathologic stage, various pathologic outcomes, adjuvant treatment, and BCR. BCR was defined as a serum PSA level ≥0.2 ng/ml on two consecutive measurements or administration of salvage treatment for a persistently rising serum PSA level. The study protocol was approved and carried out in accordance with the approved guidelines by the Institutional Review Board at the Catholic University of Korea, Seoul St. Mary’s Hospital (IRB approval No. KC14RIMI0676).

### Statistical Analysis

Statistical analysis was performed using IBM SPSS software, version 19.0 (SPSS, Inc., Chicago, Illinois, USA) and R-3.1.1 software (R Project for Statistical Computing, http://www.r-project.org/). To account for missing values in the training dataset, we imputed missing values using the random survival forest method. The missing values were inferred as weighted averages based on proximity between patients, which is the likelihood that patients are in the same leaf node of the survival trees. Continuous variables are presented as mean or median value (±standard deviation [SD]) and categorical variables are presented as proportions. Differences in the clinicopathologic features were examined using the independent t-test and chi-square test for continuous and categorical variables, respectively. BCR-free survival was estimated using Kaplan-Meier analysis and compared using the log rank test. The association between LVI and BCR was assessed using multivariate Cox proportional hazard regression analysis in a forward stepwise regression after adjusting for known important clinicopathologic features. Biopsy Gleason score and number of positive cores were excluded in advance due to the high correlations between biopsy and pathology Gleason score, and between the number of positive cores and tumor volume (0.64 and 0.33, respectively).

### Bayesian Belief Network Analysis

We used Bayesian belief network to derive the hierarchical causal relationships between BCR and predictor variables. The Bayesian belief network is a statistical graphic model that represents conditional probabilistic relationships between variables. Each node in the network represents a feature, and an edge between the nodes represents a causal dependency. If two nodes have an edge, then we can infer that the two variables have a probabilistic relationship and are more likely to occur together, thus we can identify which variables affect BCR. We learned the structure of the Bayesian belief network by hill-climbing score function using the *bnlearn* R package^28^.

### Random Survival Forest Analysis

The random survival forest is an ensemble tree method for analysis of right-censored survival data^21^. This method constructs multiple decision trees based on bootstrap data and predicts the outcome of interest (i.e. BCR) based on the majority votes of the individual decision trees. To discover which variables are the key prognostic factors to predict BCR, we ranked prognostic abilities of variables using variable importance scores. The variable importance score is defined as the increase in prediction error if the variable is randomized, providing insight into how predictive the variable is. To evaluate accuracy of the random survival forest, we used bootstrap validation with 1000 bootstraps^29^. We developed the decision trees and its importance score using training set, and validate them using test set. Training sets were randomly selected from 63.2% of patients with replacement, and test sets were un-selected (36.8% of patients), and repeated it 1000 times. This validation method has proven to be an unbiased estimate of prediction error^30^. The random survival forest was developed using the R package *RandomSurvivalForest*^21^.

## Conclusions

Our analysis of the clinicopathologic features of patients with pT3 N0 prostate cancer treated with radical prostatectomy provides evidence that LVI is one of the most powerful adverse pathologic prognostic factors of patients with pT3 N0 prostate cancer treated with radical prostatectomy. LVI status can provide clinically important prognostic information, which may aid in the decision of the postoperative management for patients with pT3 N0 prostate cancer through the proper stratification of patients, and we accordingly advocate incorporating LVI status into the present prostate cancer staging systems.

## Additional Information

**How to cite this article**: Park, Y. H. *et al.* Is lymphovascular invasion a powerful predictor for biochemical recurrence in pT3 N0 prostate cancer? Results from the K-CaP database. *Sci. Rep.* **6**, 25419; doi: 10.1038/srep25419 (2016).

## Figures and Tables

**Table 1:** Baseline demographics of the patients.

Variables	Overall	LVI status		
		LVI (+)	LVI (−)	*p-value*
Age (years)*	66.2, 67.0 (±6.5)	66.4, 67.0 (±6.1)	66.1, 66.5 (±6.6)	0.603
BMI (kg/m^2^)*	24.6, 24.6 (±2.8)	24.4, 24.2 (±2.8)	24.7, 24.8 (±2.8)	0.213
Preoperative PSA (ng/mL)*	15.7, 10.9 (±17.7)	23.0, 14.8 (±28.0)	13.6, 9.9 (±12.9)	<0.001
Total biopsy cores (n)*	11.3, 12.0 (±1.9)	11.5, 12.0 (±1.9)	11.2, 12.0 (±1.9)	0.088
Positive cores (n)*	4.9, 4.0 (±2.9)	5.6, 6.0 (±3.2)	4.4, 4.0 (±2.8)	<0.001
Biopsy Gleason score (%)				<0.001
≤6	307 (25.4)	33 (12.8)	274 (29.1)	
7 (3+4)	321 (26.5)	55 (21.4)	266 (28.2)	
7 (4+3)	197 (16.3)	43 (16.7)	154 (16.3)	
≥8	374 (30.9)	126 (49.0)	248 (26.3)	
Prostate volume (mL)*	35.5, 33.0 (±13.9)	37.2, 36.0 (± 14.5)	35.0, 32.4 (±13.6)	0.025
Tumor volume (%)				0.001
≤5 mL	568 (46.9)	101 (41.1)	467 (53.3)	
>5 mL	554 (45.8)	145 (58.9)	409 (46.7)	
Extracapsular extension (%)	1179 (97.4)	251 (96.5)	928 (97.8)	0.251
Seminal vesicle invasion (%)	297 (24.5)	118 (45.4)	178 (18.8)	<0.001
Positive surgical margin (%)	716 (59.2)	171 (65.8)	544 (57.3)	0.014
Perineural invasion (%)	1103 (91.2)	250 (96.2)	853 (89.9)	0.002
Pathologic Gleason score (%)				<0.001
≤6	85 (7.0)	4 (1.5)	81 (8.6)	
7 (3+4)	452 (37.4)	52 (20.1)	400 (42.2)	
7 (4+3)	350 (28.9)	95 (36.7)	255 (26.9)	
≥8	320 (26.4)	108 (41.7)	211 (22.3)	

^*^Values are expressed as mean, median (±SD)

LVI, lymphovascular invasion; BMI, body mass index; PSA, prostate-specific antigen.

**Table 2:** Univariate and multivariate Cox proportional hazard regression analysis of clinicopathologic features for biochemical recurrence.

Factors	Univariate analysis			Multivariate analysis		
	HR	95% CI	*p-value*	HR	95% CI	*p-value*
Age (years)	1.009	0.992–1.025	0.313	1.000	0.982–1.018	0.982
BMI (kg/m^2^)	0.972	0.936–1.011	0.154	0.965	0.925–1.006	0.090
Preoperative PSA (ng/mL)	1.022	1.009–1.036	<0.001	1.013	1.008–1.216	0.018
Prostate volume (mL)	1.004	0.996–1.012	0.311	0.997	0.987–1.006	0.484
Tumor volume (> 5mL)	1.912	1.530–2.391	<0.001	1.506	1.191–1.903	0.001
Extracapsular extension	0.943	0.517–1.720	0.847	1.378	0.709–2.679	0.345
Seminal vesicle invasion	2.348	1.890–2.917	<0.001	1.588	1.245–2.026	<0.001
Positive surgical margin	1.659	1.323–2.081	<0.001	1.171	0.913–1.500	0.214
Lymphovascular invasion	2.038	1.635–2.541	<0.001	1.357	1.064–1.730	0.014
Perineural invasion	1.392	1.002–1.684	0.023	1.278	1.039–1.896	0.230
Pathologic Gleason score						
≤6	Reference			Reference		
7 (3+4)	1.080	0.631–1.849	0.778	1.041	0.575–1.881	0.895
7 (4+3)	2.087	1.229–3.546	0.006	1.731	1.001–3.119	0.048
≥8	4.247	2.528–7.137	<0.001	3.127	1.744–5.606	<0.001
Adjuvant treatment	2.301	1.216–3.482	0.002	1.562	0.913–2.896	0.231

HR, hazard ratio; CI, confidence interval; BMI, body mass index; PSA, prostate-specific antigen.
